# V_3_S_4_ Nanosheets Anchored on N, S Co-Doped Graphene with Pseudocapacitive Effect for Fast and Durable Lithium Storage

**DOI:** 10.3390/nano9111638

**Published:** 2019-11-18

**Authors:** Naiteng Wu, Di Miao, Xinliang Zhou, Lilei Zhang, Guilong Liu, Donglei Guo, Xianming Liu

**Affiliations:** 1 Key Laboratory of Function-oriented Porous Materials, College of Chemistry and Chemical Engineering, Luoyang Normal University, Luoyang 471934, China; miao990831@163.com (D.M.); chevasz@163.com (X.Z.); zhanglilei@outlook.com (L.Z.); glliu@tju.edu.cn (G.L.); gdl0594@163.com (D.G.); 2 College of Materials and Chemical Engineering, China Three Gorges University, Yichang 443002, China

**Keywords:** V_3_S_4_ nanosheets, pseudocapacitive, N, S co-doped graphene, lithium ion batteries

## Abstract

Construction of a suitable hybrid structure has been considered an important approach to address the defects of metal sulfide anode materials. V_3_S_4_ nanosheets anchored on an N, S co-coped graphene (VS/NSG) aerogel were successfully fabricated by an efficient self-assembled strategy. During the heat treatment process, decomposition, sulfuration and N, S co-doping occurred. This hybrid structure was not only endowed with an enhanced capability to buffer the volume expansion, but also improved electron conductivity as a result of the conductive network that had been constructed. The dominating pseudocapacitive contribution (57.78% at 1 mV s^−1^) enhanced the electrochemical performance effectively. When serving as anode material for lithium ion batteries, VS/NSG exhibits excellent lithium storage properties, including high rate capacity (480 and 330 mAh g^−1^ at 5 and 10 A g^−1^, respectively) and stable cyclic performance (692 mAh g^−1^ after 400 cycles at 2 A g^−1^).

## 1. Introduction

Lithium ion batteries (LIBs) are the dominant energy storage device in the field of mobile devices. A high capacity, high rate, long cyclic life, and safety are the critical elements for their large-scale application [1]. Traditional graphite anodes, on the other hand, could not meet the above requirements. Numerous novel candidates, such as transition metal oxide, sulfide, alloy, and silicon, have been explored to replace the commercial graphite anode material.

Among these anode materials, the earth-abundant and high capacity and transition of multiple valence vanadium-based materials are considered as the most promising candidate for the advanced LIBs [2]. V_2_O_3_ [3,4], VO_2_ [5], V_6_O_13_ [6]_,_ and other metal vanadium oxide composites [7,8,9,10] have been reported in previous works. However, inferior electric conductivity is a common drawback for these vanadium oxides, resulting in insufficient rate performances [2]. For vanadium sulfides, the moderate V–S bond would decrease the electrochemical polarization and benefit the intercalation and deintercalation of Li^+^ or other alkali metal ions, which would further result in improved electrochemical properties [7]. However, bulk metal sulfides usually suffer from large volumetric expansion (resulting in the obvious capacity fading) and sluggish electron transport kinetics (the cause of the inferior rate property) [8,9]. Construction of nanostructures and composites with conductive substrates are considered an efficient route to overcome the drawbacks of bulk vanadium sulfides. For example, flower-like VS_2_ nanosheets [10], VS_4_ microsphere@PANI [11], VS_4_ nanoparticles@CNTs [12], VS_4_ nanowire [13], nanosheets, nanoparticles [14] anchored on graphene, V_2_S_3_ microsphere@C [15], and V_5_S_8_–/graphite hybrid nanosheet [16,17] anodes delivered the improved Li^+^/Na^+^ storage performances. According to the above works, VS_4_ microsphere@PANI delivered a capacity of 755 mAh g^−1^ at the 50th cycle under a current density of 0.1 A g^−1^ [11], and the VS_4_/graphene composite exhibited a capacity of 954 mAh g^−1^ at the end of 100 cycles under the same current density [18]. The durable lithium storage under high current density would not be obtained. Furthermore, toxic thioacetamide and hydrogen sulfide are always used as sulfide sources in these synthesis processes. The green synthesis route is still a challenge for the application of vanadium sulfides anode materials.

In this work, V_3_S_4_ nanosheets anchored on N, S co-doped graphene have been fabricated through a facile and green method. NH_4_VO_3_ and (NH_2_)_2_CS were used as vanadium and sulfide sources, respectively. V_3_S_4_ nanosheets shorten the transmission path of both electron and Li ions. Meanwhile, the formed N, S co-doped graphene aerogel not only endow the as-prepared anode with the enhanced capability to buffer the volume expansion but also construct a conductive network to improve electron conductivity. Moreover, the dominating pseudocapacitive contribution (57.78% at 1 mV s^−1^) is effective for improving the electrochemical performances. When serving as an anode material for LIBs, V_3_S_4_ nanosheet anchored on N, S-doped graphene exhibits a high rate capacity (480 and 330 mAh g^−1^ at 5 and 10 A g^−1^, respectively) and stable cyclic performance at high rate (692 mAh g^−1^ after 400 cycles at 2 A g^−1^).

## 2. Materials and Methods

### 2.1. Materials Synthesis

Synthesis of V_3_S_4_/N, S-rGO (reduced graphene oxides) nanosheets. In a typical synthesis, 2.5 mmol NH_4_VO_3_ and 1.4 g (NH_2_)_2_CS were dissolved in 20 mL formed GO (graphene oxides) aqueous solution (5 mg mL^−1^) at 60 °C. Then, the mixed solution was transferred into a 25 mL Teflon-lined stainless-steel autoclave and heated at 180 °C for 20 h in an oven. After hydrothermal treatment, the formed hydrogel was freeze-dried and annealed at 600 °C for 5 h under Ar atmosphere to obtain the final samples (denoted as VS/NSG).

### 2.2. Materials Characterization

X-ray diffraction (XRD, Bruker D8 with Cu Kα radiation, Billerica, MA, USA) analysis was carried out to determine the crystal structure of the samples. The morphology and the structure of the samples were investigated by SEM (SEM, Sigma 500, Zeiss, Oberkochen, Germany) and transmission electron microscopy (TEM, JEOL JEM2100, Tokyo, Japan). X-ray photoelectron spectroscopy (XPS, EscaLab 250Xi, Shanghai, China) was performed to determine the valence state of the products. The graphitic degree of reduced graphene oxide was investigated by Raman spectrometer with an excitation laser beam wavelength of 633 nm (HORIBA Jobin-Yvon, LabRAM Aramis, Kyoto, Japan). To investigate the content of the coating carbon, thermal gravimetric analysis was conducted on a thermal analyzer (SII TG/DTA6300, Tokyo, Japan) in air at a heating rate of 5 °C min^−1^.

### 2.3. Electrochemical Measurements

V_3_S_4_ nanosheet anchored on N, S-doped graphene was assembled CR2032 half-coin cells to evaluate its electrochemical performance. The assembly processes were similar to our previous works [19,20]. Typically, the mass loading of active material is about 1.2–1.3 mg cm^−2^. The charge–discharge processes were operated at the voltage range of 0.005–3 V using Neware CT3008 (Neware, Shenzhen, China). Cyclic voltammetry measurements (CV, at the scanning rate from 0.1 to 1.0 mV s^−1^) and electrochemical impedance spectroscopy (EIS, in the frequency range from 100,000–0.01 Hz with an AC amplitude of 5 mV) were conducted on the Parstat 4000+ workstation (Princeton Applied Research).

## 3. Results

**Figure 1:**
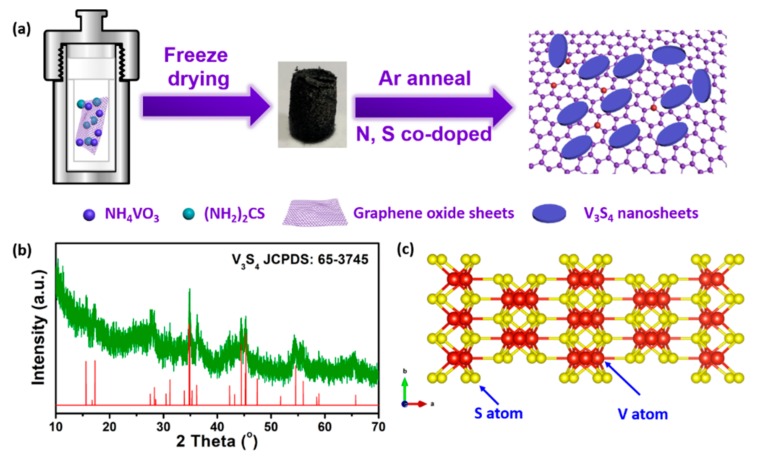
(**a**) Schematic illustration of preparation process of VS/NSG; (**b**) XRD pattern of as-prepared VS/NSG; (**c**) the structure of V_3_S_4_ along the c-axis.

The preparation process is illustrated in Figure 1a. XRD was firstly conducted to investigate the crystal phase of as-prepared sample. As shown in Figure 1b, most of diffraction peaks of as-prepared sample can be indexed as the standard monoclinic V_3_S_4_ phase (JCPDS no. 65-3745). The low intensity of diffraction peaks would be ascribed to the crystallinity of the nanosheet and graphene substrate. In Figure 1c, there is an obvious interlayer spacing along the *c*-axis, which is convenient for the storage of lithium ions. The content of graphene substrate was exposed by TG analysis. After the heat treatment process in air atmosphere, VS/NSG would be decomposed and oxidized to V_2_O_5_. In this process, the change of weight is about −3%. According to the results of TG analysis (Figure S1), the content of graphene substrate is about 38 wt %. As shown in Figure S2, VS/NSG exhibits two typical D and G bands at around 1360 and 1585 cm^−1^, corresponding to *sp*^3^-type disordered carbon and *sp*^2^-type ordered graphitic carbon [21,22]. The intensity ratio of G and D band (*I*_G_/*I*_D_ = 1.08) suggests the more graphitic carbon in the graphene substrate.

**Figure 2:**
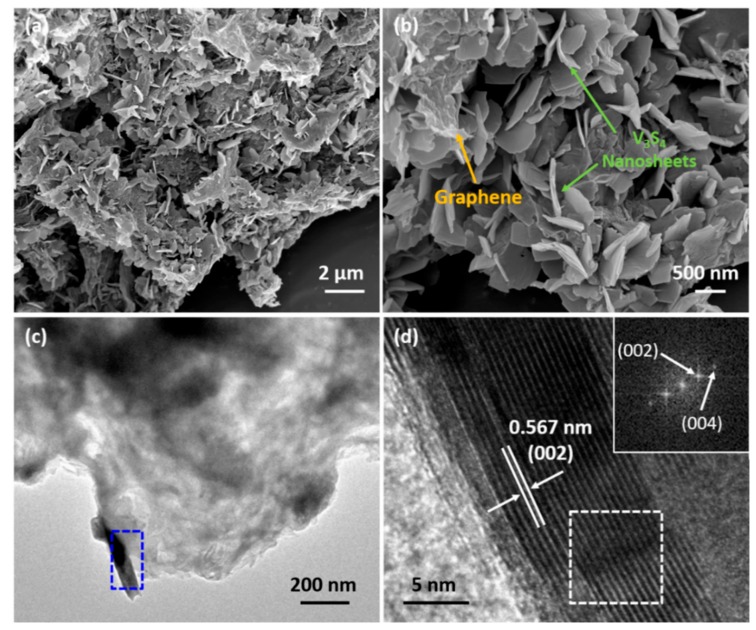
(**a**,**b**) SEM images of as-prepared VS/NSG at different magnifications; (**c**,**d**) TEM image, HRTEM image, and FFT patterns in the marked white box of VS/NSG.

SEM and TEM were used to characterize the detail morphology and structure of VS/NSG. In the panoramic SEM image (Figure 2a), VS/NSG exhibits morphology typical of nanosheet and graphene composites. Numerous V_3_S_4_ nanosheets anchor onto the graphene substrate to ensure their electronic conductivity. As shown in the magnified SEM image (Figure 2b), the grown nanosheets display a smooth surface with particles of about 500–1500 nm in diameter and only 70–80 nm in width. The composite structure of VS/NSG can be further observed in Figure 2c. The HRTEM (high-resolution transmission electron microscopy) image (Figure 2d) selected from the blue box in Figure 2c displays the clear lattice fringes. Assisted by the fast Fourier transform patterns (FFT, inset of Figure 2d) calculated from the white box region, the interplanar distance can be measured of about 0.567 nm and assigned to the (002) facet of V_3_S_4_.

**Figure 3:**
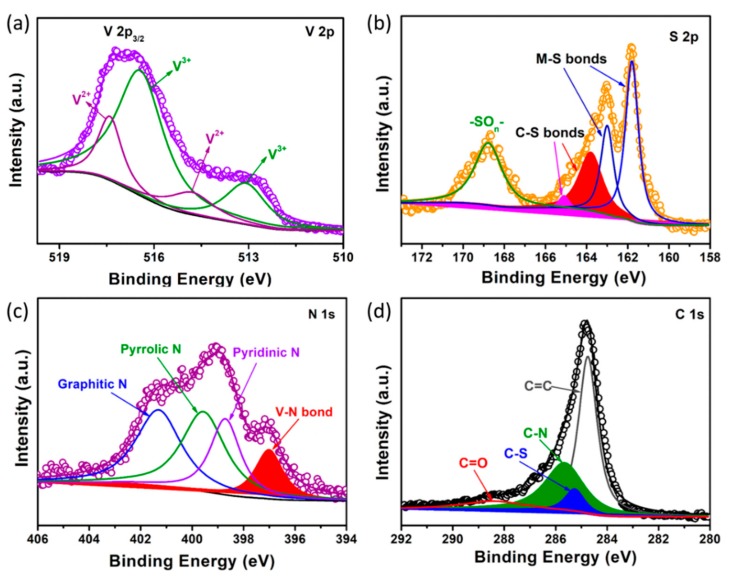
XPS survey (**a**) V 2p, (**b**) S 2p, (**c**) N 1s, and (**d**) C 1s of as-prepared VS/NSG.

XPS measurements were carried out to ascertain the surface chemical characteristics of VS/NSG. As shown in Figure S3, the survey spectrum exhibits the peaks of V, S, C, N, and O elements. The high-resolution of V 2p spectrum (Figure 3a) displays four components, corresponding to the V^2+^ and V^3+^, respectively [7,23]. In Figure 3b, S 2p spectrum exhibits obvious three peaks, which can be assigned to the typical M–S (M = metal), C–S, and common S–O bonds [8,9,24,25]. However, the S 2p spectrum of VS/NSG suggested that the S-doped graphene had been affected by the presence of sulfides. To address this confusion, the graphene substrate formed by the acid-treated VS/NSG was also characterized. As shown in Figure S4, the high-resolution S 2p spectrum of acid-washed sample also displays obvious C–S bonds, confirming that the sulfur had been doped into the graphene. Impressively, the existence of the C–S bond suggests the presence of sulfur covalently bonded to graphene in a heterocyclic configuration [26]. As shown in Figure 3c, four peaks represent the graphitic N, pyrrolic N, pyridinic N, and V–N bond, respectively [27,28,29]. This result is good verification of the expectation when alien atoms are doped into graphene. Moreover, the obvious V–N bond peak located at 397 eV demonstrates the formation of VN on the surface of VS/NSG, which would enhance the conductivity of as-prepared sample. Moreover, the nitrogen content is about 8.86 at %. The high content of doped nitrogen would be beneficial regarding the improvement of conductivity. The presence of C–N and C–S peaks in the C 1s high-resolution spectrum further confirm the conclusion that N, S co-doped graphene was formed (Figure 3d) [29].

**Figure 4:**
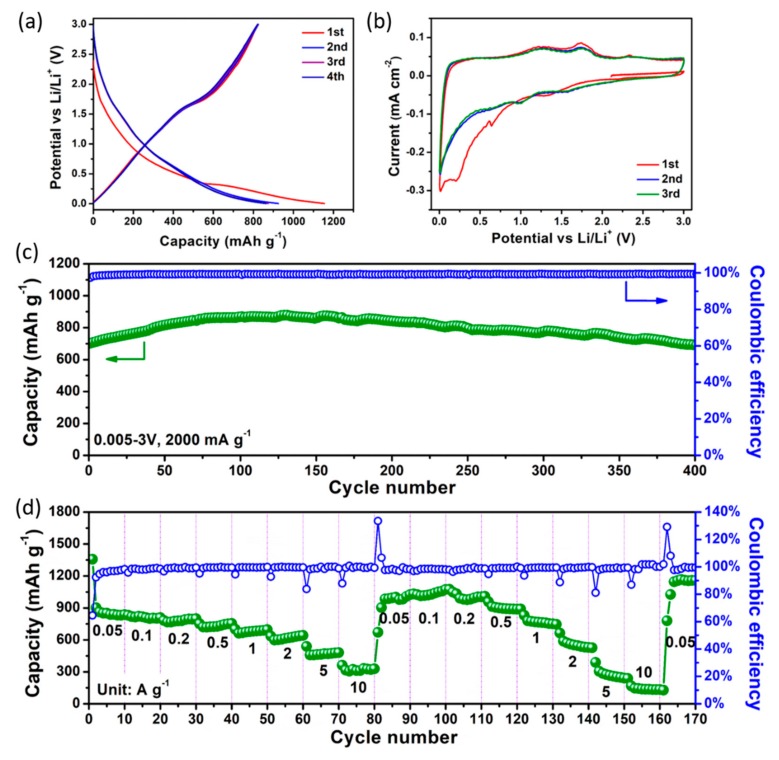
(**a**) The initial discharge–charge curves of VS/NSG at 0.05 A g^−1^; (**b**) CV curves of VS/NSG at a scan rate of 0.1 mV s^−1^; and (**c**) the cyclic stability and (**d**) rate capability of VS/NSG electrode.

The lithium storage performances of VS/NSG were evaluated by CR2032 half-coin cells. As shown in Figure 4a, in the voltage range of 0.005–3 V under 50 mA g^−1^, VS/NSG electrode delivers the initial discharge and charge capacity of 1150 and 825 mAh g^−1^, respectively. The initial coulombic efficiency (ICE) of V_3_S_4_ nanosheet is about 71.7%, which is much higher than that of other transition metal oxides and sulfides, such as SnO_2_ [30], CoO [31], Co_3_O_4_ [32], V_2_O_3_ [4], and VS_4_ [13]. After the initial cycle, the overlapped discharge–charge curves suggest the well reversibility of as-prepared VS/NSG. Cyclic voltammetry (CV) were used to investigate the lithium storage behavior of V_3_S_4_ nanosheet. The cathodic peaks located at 1.29, 0.64, and 0.22 V in the first sweep can be assigned to the behavior of Li^+^ insertion into V_3_S_4_ interlayer stepwise and formation of solid electrolyte interface (SEI) layer, respectively [12,13,33]. At the following anodic sweep, the anodic peaks at 1.25, 1.72, and 2.33 V correspond to the delithiation process of Li*_x_*V_3_S_4_ and formation of V_3_S_4_ [7,17]. The overlapped CV curves at the second and third cycle indicate the good reversibility and cyclic stability of VS/NSG electrode. As shown in Figure 4c, the cyclic stability of VS/NSG were tested under a current density of 2000 mA g^−1^. At this high current density, VS/NSG electrode delivers an excellent cycling stability. After 400 monotonous cycles, as high as 692 mAh g^−1^ discharge capacity can be retained. The level of coulombic efficiency, being close to 100%, suggests the high reversibility of VS/NSG during the monotonous cycles. As shown in Figure S5, the counterpart of VS/NSG, which without the N, S co-doped graphene substrate delivers the discharge capacity of 856 mAh g^−1^ with the ICE only about 60.1%. In the following cyclic test, the bare cell exhibits obvious capacity fading. In the following rate tests, VS/NSG electrode delivers a high reversible discharge capacity from 0.05 to 10 and back to 0.05 A g^−1^, which reach 480 and 330 mAh g^−1^ at 5 and 10 A g^−1^, respectively. Particularly, in the subsequent repetition of rate evaluations, the discharge capacity of VS/NSG at low current densities exhibits an obvious increasing tendency. This phenomenon would be attributed to the increased Li^+^ storage ability at the enhanced area of electrolyte and electrode interface. When the current density is back to 0.05 A g^−1^, a high discharge capacity of 1150 mAh g^−1^ can be retained, indicating the superior rate capability of VS/NSG electrode.

**Figure 5:**
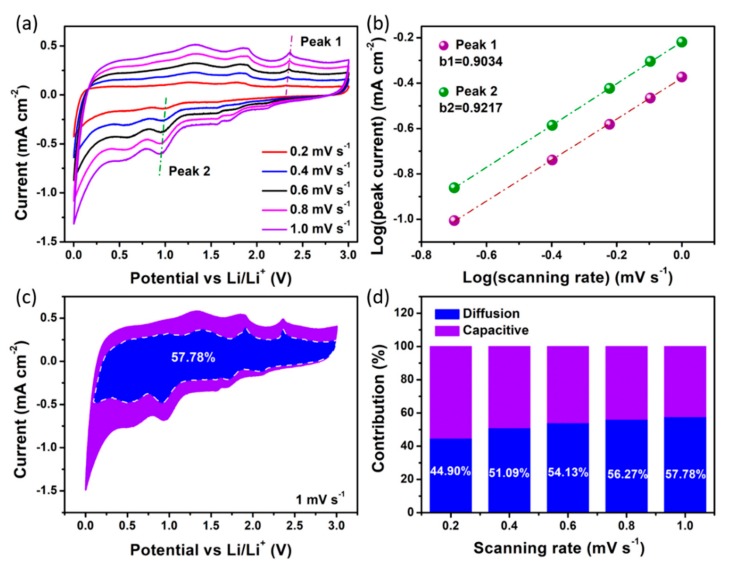
Kinetic analyses of VS/NSG electrode. (**a**) CV curves at different scanning rates; (**b**) log(*i*) vs. log (*v*) plots at different peaks; (**c**) capacitive contribution (blue region) to the charge storage at 1 mV s^−1^; (**d**) the contribution ratio of pseudocapacitive and diffusion-controlled current at different scanning rates.

Kinetic analyses based on the CV curves were carried out to reveal the potential reasons for the excellent cyclic stability of VS/NSG electrode. At different scanning rates ranging from 0.2 to 1.0 mV s^−1^ (Figure 5a), the CV curves displayed a similar and broadened shape. The overall charge storage, which is revealed by the integral area of CV curve, could be attributed to by the surface-induced capacitive and diffusion–insertion process [19,34]. Based on the CV curves at different scanning rates and Equation (1) [35], the charge storage mechanism of electrode materials could be studied.

*i*(*V*) = *av^b^* (1)

According to the previous reports, the value of *b* in this equation is an indicator to investigate the mechanism of charge storage [20,35]. As shown in Figure 5b, the value of *b* based on peak 1 and peak 2 is 0.9034 and 0.9217, respectively, which are fitted by the slopes of log*v* and log*i* (*v* means the scanning rate and *i* represents the peak current). These values mean that surface-induced capacitive would be a dominator in VS/NSG electrode charge storage system. The ratio of surface-induced capacitive in the whole charge storage can be further calculated using Equation (2).

*i*(*V*) = *k*_1_*v* + *k*_2_*v*^0.5^ (2)

The integral area of *k*_1_*v* and k_2_*v*^0.5^ means the surface-induced capacitive and diffusion–insertion process, respectively [36,37]. Furthermore, the values of *k*_1_ at different potentials are the slope of the function of *i*(V)/*v*^0.5^~*v*^0.5^ in a series of CV analyses. Based on the various *k*_1_ at different potential, the profile of surface-induced capacitive under a certain scanning rate could be depicted. As shown in Figure 5c, the blue area represents the charge storage contribution of surface-induced capacitive. The integral area ratio of blue and violet area is 57.78%, meaning the pseudocapacitive contribution is 57.78% at the scanning rate of 1.0 mV s^−1^. In addition, the surface-induced capacitive profiles and pseudocapacitive contributions from 0.2 to 0.8 mV s^−1^ are depicted in Figure S6 and Figure 5d. The pseudocapacitive contribution is enhanced gradually with the increasing scanning rate, and the ratio at 0.2, 0.4, 0.6, and 0.8 mV s^−1^ is 44.90%, 51.09%, 54.13%, and 56.27%, respectively. Dominant pseudocapacitive effect at fast scanning rate endow the VS/NSG electrode with excellent cyclic stability and rate capability. Moreover, Nyquist plots of VS/NSG electrode at the 3rd and 50th cycle are displayed in Figure S7. Clearly, the smaller semicircle of VS/NSG after 50 cycles, meaning a smaller resistance of charge transfer (*R*_st_, an indicator to the degree of side reaction [38,39,40]), reveals better conductivity due to the increased compatibility of electrode material with electrolyte [41]. The above kinetic analyses indicate that the V_3_S_4_ nanosheets anchored on N, S co-doped graphene would induce the dominating pseudocapacitive effect and restrain the increasing of *R*_st_.

## 4. Conclusions

In summary, the fabrication of V_3_S_4_ nanosheets anchored on N, S co-doped graphene by this universal and green method exhibits excellent rate capability and cyclic stability. At the current density of 5 and 10 A g^−1^, the as-prepared VS/NSG delivers a high discharge capacity of 480 and 330 mAh g^−1^, respectively. At the end of 400 cycles under 2 A g^−1^, 692 mAh g^−1^ also can be retained. The superior electrochemical performances of VS/NSG benefited by the formed N, S co-doped graphene aerogel, not only endow it with an enhanced capability to buffer the volume expansion, but also a constructed conductive network that improves its electron conductivity. Moreover, the dominant pseudocapacitive contribution at fast scanning rate and the decreasing resistance of charge transfer also guarantee fast and durable Li^+^ storage performance.

## Supplementary Materials

The following are available online at https://www.mdpi.com/2079-4991/9/11/1638/s1, Figure S1: TG curve of V_3_S_4_/N, S-rGO nanosheets under air atmosphere, Figure S2: Raman spectrum of VS/NSG sample, Figure S3: XPS spectrum of as-prepared V_3_S_4_/N, S-rGO nanosheets, Figure S4: XPS S 2p spectrum of VS/NSG treated after acid, Figure S5: (a) Initial discharge-charge curves and (b) cyclic performance of V_3_S_4_ counterpart under 0.05 and 0.5 A g-1, respectively. Figure S6: Capacitive current contribution (blue region) to the charge storage at (a) 0.2, (b) 0.4, (c) 0.6 and (d) 0.8 mV s-1 of V_3_S_4_/N, S-rGO nanosheets, Figure S7: Nyquist plots of V_3_S_4_/N, S-rGO nanosheets cell at 3nd and after 50 cycles.
