# Supplementary material for: V3S4 Nanosheets Anchored on N, S Co-Doped Graphene with Pseudocapacitive Effect for Fast and Durable Lithium Storage
Source: Nanomaterials (Basel). 2019 Nov 18;9(11):1638. doi: 10.3390/nano9111638 (PMC6915494; doi:10.3390/nano9111638)
Supplement: Supplementary file 1 [file nanomaterials-09-01638-s001.pdf]

Supplementary Materials

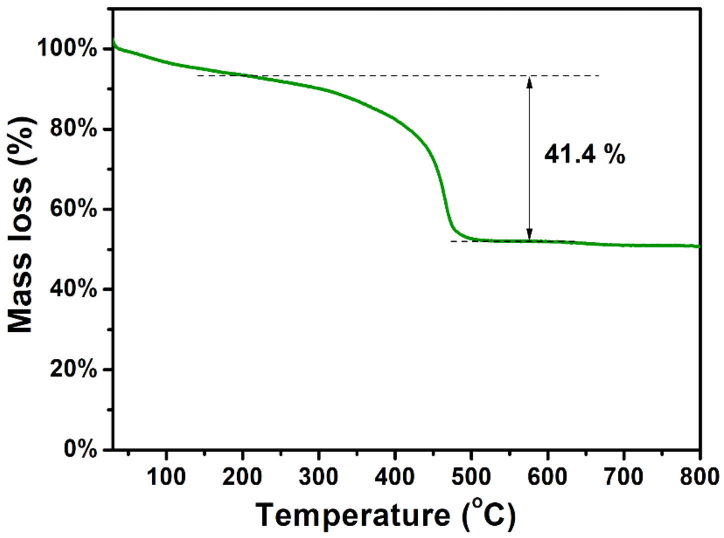

Figure S1: TG curve of V_3S_4/N, S-rGO nanosheets under air atmosphere.

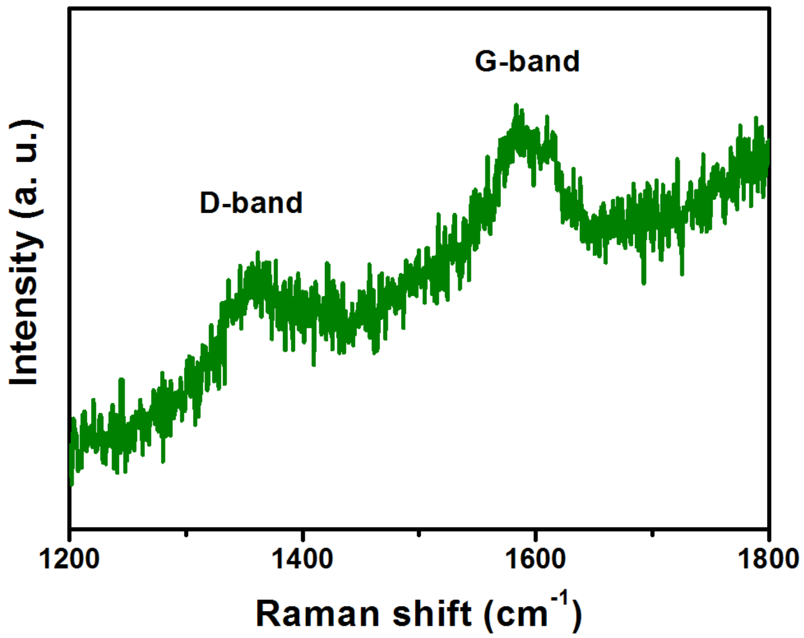

Figure S2: Raman spectrum of VS/NSG sample.

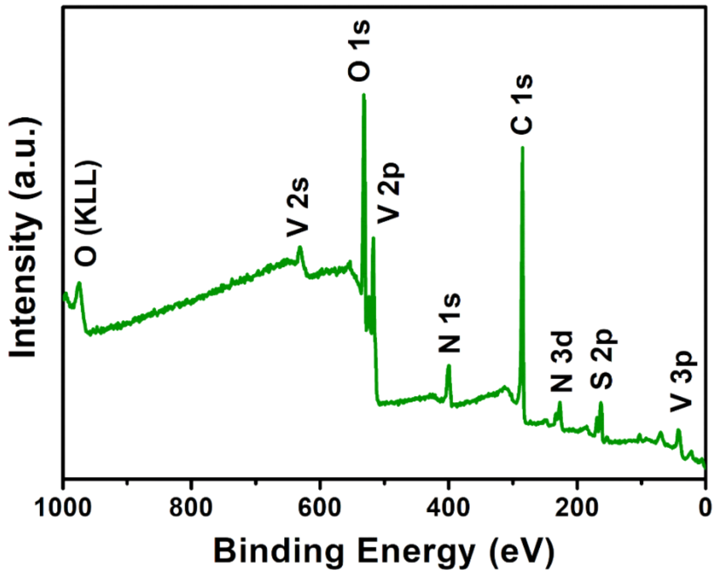

Figure S3: XPS spectrum of as-prepared V_3S_4/N, S-rGO nanosheets.

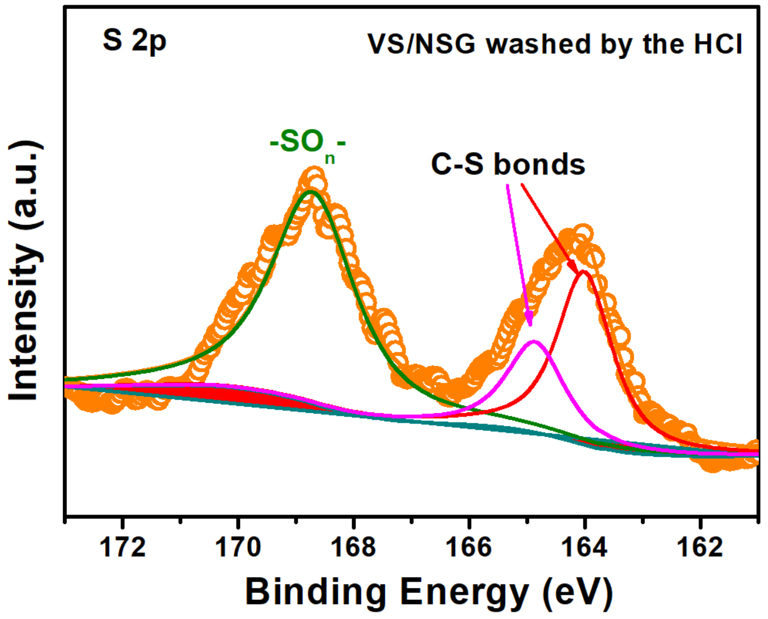

Figure S4: XPS S 2p spectrum of VS/NSG treated after acid.

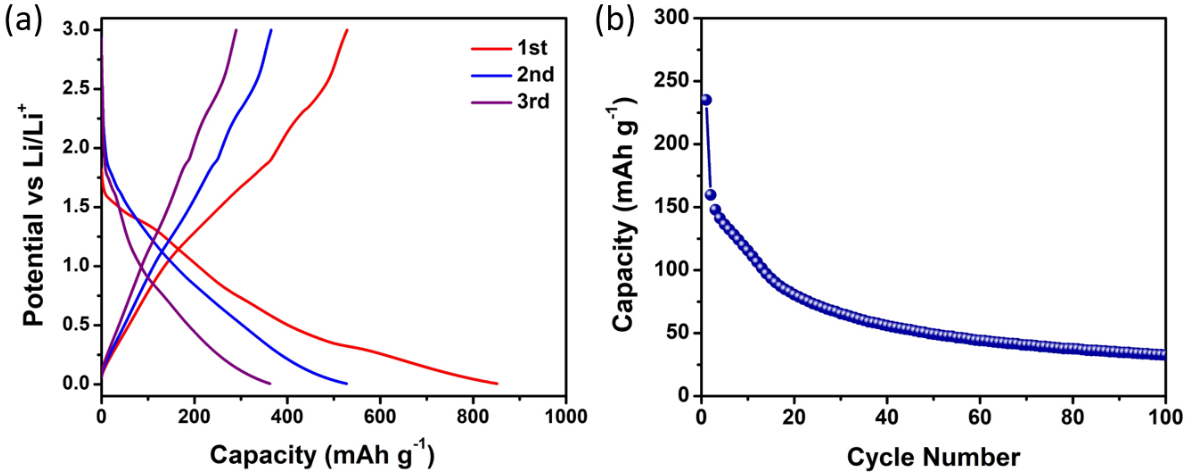

Figure S5: (a) Initial discharge-charge curves and (b) cyclic performance of V_3S_4 counterpart under 0.05 and 0.5 A g^{-1}, respectively.

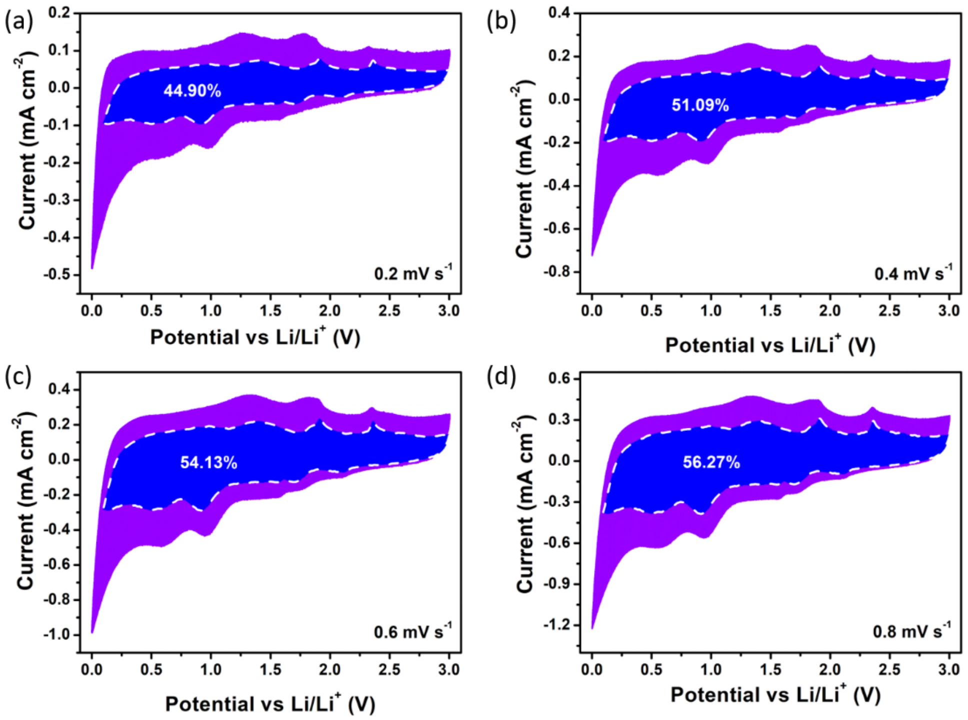

Figure S6: Capacitive current contribution (blue region) to the charge storage at (a) 0.2, (b) 0.4, (c) 0.6 and (d) 0.8 mV s^{-1} of V_3S_4/N, S-rGO nanosheets.

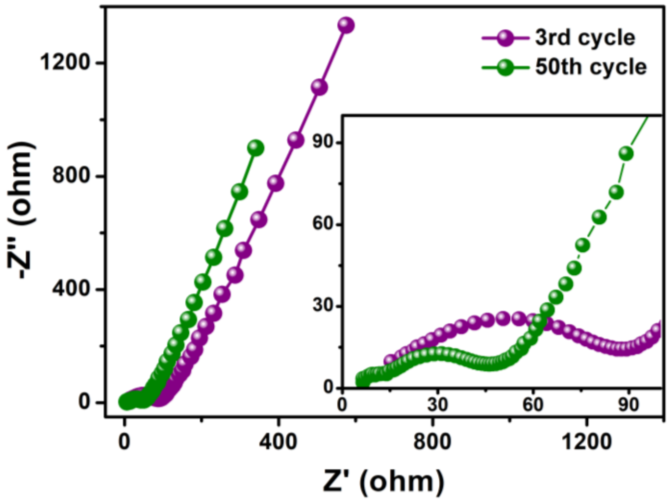

Figure S7: Nyquist plots of V_3S_4/N, S-rGO nanosheets cell at 3nd and after 50 cycles.
